# *Trypanosoma cruzi* Infection Induces Cellular Stress Response and Senescence-Like Phenotype in Murine Fibroblasts

**DOI:** 10.3389/fimmu.2018.01569

**Published:** 2018-07-09

**Authors:** Kamila Guimarães-Pinto, Danielle Oliveira Nascimento, Antonia Corrêa-Ferreira, Alexandre Morrot, Celio G. Freire-de-Lima, Marcela F. Lopes, George A. DosReis, Alessandra A. Filardy

**Affiliations:** ^1^Instituto de Biofísica Carlos Chagas Filho, Universidade Federal do Rio de Janeiro, Rio de Janeiro, Brazil; ^2^Departamento de Imunologia, Instituto de Microbiologia Paulo de Góes, Universidade Federal do Rio de Janeiro, Rio de Janeiro, Brazil; ^3^Faculdade de Medicina, Universidade Federal do Rio de Janeiro, Rio de Janeiro, Brazil; ^4^Laboratório de Imunoparasitologia, Instituto Oswaldo Cruz, Fundação Oswaldo Cruz, Rio de Janeiro, Brazil; ^5^Instituto Nacional para Pesquisa Translacional em Saúde e Ambiente na Região Amazônica, Conselho Nacional de Desenvolvimento Científico e Tecnológico, Rio de Janeiro, Brazil

**Keywords:** Chagas disease, *Trypanosoma cruzi*, senescent-like, senescence-associated β-galactosidase, senescence-associated secretory phenotype, reactive oxygen species, deferoxamine

## Abstract

*Trypanosoma cruzi* infects and replicates within a wide variety of immune and non-immune cells. Here, we investigated early cellular responses induced in NIH-3T3 fibroblasts upon infection with trypomastigote forms of *T. cruzi*. We show that fibroblasts were susceptible to *T. cruzi* infection and started to release trypomastigotes to the culture medium after 4 days of infection. Also, we found that *T. cruzi* infection reduced the number of fibroblasts in 3-day cell cultures, by altering fibroblast proliferation. Infected fibroblasts displayed distinctive phenotypic alterations, including enlarged and flattened morphology with a nuclei accumulation of senescence-associated heterochromatin foci. In addition, infection induced an overexpression of the enzyme senescence-associated β-galactosidase (SA-β-gal), an activation marker of the cellular senescence program, as well as the production of cytokines and chemokines involved with the senescence-associated secretory phenotype (SASP) such as IL-6, TNF-α, IL-1β, and MCP-1. Infected fibroblasts released increased amounts of stress-associated factors nitric oxide (NO) and reactive oxygen species (ROS), and the treatment with antioxidants deferoxamine (DFO) and *N*-acetylcysteine reduced ROS generation, secretion of SASP-related cytokine IL-6, SA-β-gal activity, and parasite load by infected fibroblasts. Taken together, our data suggest that *T. cruzi* infection triggers a rapid cellular stress response followed by induction of a senescent-like phenotype in NIH-3T3 fibroblasts, enabling them to act as reservoirs of parasites during the early stages of the Chagas disease.

## Introduction

The intracellular protozoan parasite *Trypanosoma cruzi* is the etiological agent of Chagas disease, the main endemic parasitic disease of Latin America, and a public health issue in non-endemic regions, including USA (1–3). Natural route of infection occurs when a triatomine insect vector deposit infective metacyclic trypomastigotes with its feces on the host’s skin during blood meal. The parasites penetrate the skin and dermis by small lesions caused by wound scratching (4, 5). Trypomastigotes invade immune and non-immune host cells, where they are converted into the replicative amastigote form. After several rounds of replication, amastigotes differentiate into highly motile infective trypomastigotes that are released in the intercellular spaces to disseminate the infection to other cells (6, 7). The early parasite interactions with host cells may determine the outcome of *T. cruzi* infection. However, most of the studies are focused on macrophages and cardiomyocytes, since they are responsible for triggering immune responses and cardiac lesions in Chagas disease, respectively (8, 9). Nonetheless, before the parasites gain access to these cell types, they need to interact/invade epithelial cells and fibroblasts that compose epithelial/mucous barriers to insure dissemination and the establishment of chronic infection (10–12). Particularly, *T. cruzi* has the ability to non-selectively infect a wide range of cell types, which is probably due to its ability to simultaneously express several surface glycoproteins, and interact with several mammalian receptors, including toll-like receptors, TGF- and EGF-receptors, and tyrosine kinases receptors; both factors are required for optimal parasite adhesion, penetration, and transit through host cell parasitophorous vacuoles in order to establish an intracellular infection (8, 11, 13, 14). Several *in vitro* studies have shown that trypomastigotes infect epithelial cells and fibroblasts (6, 11, 15–17); and an interesting histopathological investigation, that analyzed placentas from mothers who gave birth to babies congenitally infected with *T. cruzi*, has shown parasites in fibroblasts and macrophages of chorion, membranes, and chorionic plate, mainly in the area of membrane insertion (18).

*Trypanosoma cruzi* employs a number of strategies to evade the immune responses and prevail itself in the infected hosts. Regulating host cell cycle is one of the mechanisms used by many intracellular pathogens, including *T. cruzi*, to facilitate its replication and perpetuity within the organism (19–22). It has been shown that resistance and susceptibility to *T. cruzi* infection is cyclic and varies accordingly to cell cycle phase (23). In addition, it was reported that *T. cruzi* infection impedes cell cycle progression (22), and induces an upregulation of several genes involved in cell cycle control, suppressing proliferation in the host non-immune cells (11).

Suppression of cell proliferation is the most prominent feature of cellular senescence, a complex stress response, in which senescent cells undergo broad morphological and phenotypic changes (24). Senescent cells display increased cell size and flat morphology with a nuclei accumulation of senescence-associated heterochromatin foci (SAHF) (25, 26). Besides that, senescent cells are characterized by increased secretion of many factors including senescence-associated β-galactosidase (SA-β-gal) (27, 28), as well as proinflammatory cytokines/chemokines, and NO, which characterize senescence-associated secretory phenotype (SASP) (29, 30). SASP cytokines/chemokines include IL-6, TNF-α, IL-1β, and MCP-1 (30, 31), which have effects in the tissue microenvironment, propagating the stress response and communicating with neighboring cells. Cellular senescence has been associated with the process of aging (32), during embryogenesis (33), and diseases such as cancer (34, 35) and infectious diseases (36–39). Particularly, it has been already described that chronic immune activation during Chagas disease results in CD4^+^ and CD8^+^ T cells immune senescence due to cell exhaustion by the *T. cruzi* persistence in the host (40, 41). In addition, cardiomyocytes infected with *T. cruzi* have enhanced reactive oxygen species (ROS) production, which leads to DNA damage (42). However, whether DNA damage induces senescence in Chagas disease or even whether senescence is induced in other non-immune cell types during the acute phase of infection remains to be addressed.

Here, we investigated early cellular responses to *T. cruzi* infection that may be relevant to the establishment of chronic disease by using the murine fibroblast cell NIH-3T3. We found that *T. cruzi* infection reduced fibroblasts proliferation and altered their morphology to enlarged and flattened cells, and promoted nuclei accumulation of SAHF. Concomitantly, infected fibroblasts had increased SA-β-gal activity, and production of cytokines involved with SASP such as IL-6, TNF-α, and IL-1β, as well as the chemokine MCP-1. In addition, we observed that *T. cruzi*-infected fibroblasts released increased levels of NO and ROS, and the addition of antioxidants DFO and *N*-acetylcysteine (NAC) reduced ROS generation, decreased the secretion of SASP-related cytokine IL-6, and SA-β-gal activity, and reduced the parasite load by infected fibroblasts. Taken together, our findings suggest that *T. cruzi* infection triggers a rapid cellular stress response in NIH-3T3 fibroblasts, culminating in an induction of a senescent-like phenotype that enables these cells to act as reservoirs of parasites, during the early stages of the Chagas disease.

## Materials and Methods

### Mammalian Cell Culture and Parasites

Murine NIH-3T3 fibroblasts (Instituto Nacional de Controle de Qualidade em Saúde; Fiocruz, Brazil) were maintained in Dulbecco’s modified Eagle’s medium (Gibco) supplemented with 2 mM glutamine, 5 × 10^−5^ M 2-Mercaptoethanol (2-ME), 10 µg/mL gentamicin, 1 mM sodium pyruvate, and 0.1 mM MEM non-essential amino acids plus 10% fetal bovine serum (FBS) (all from Gibco) at 37°C in a 5% CO_2_ atmosphere. Tissue culture-derived *T. cruzi* trypomastigotes (Dm28c strain) were obtained from the supernatant of infected LLC-MK2 (Rhesus monkey kidney epithelial cells; ATCC, Manassas, VA, USA), 5–9 days post-infection (p.i.) by weekly passages (43). All experiments were performed under biosafety level 2.

### Fibroblast Infection and Parasite Load

NIH-3T3 fibroblasts monolayers (5 × 10^4^ cells/well in 24-well plates) were seeded and after 2 h, adherent cells were infected with tissue culture-derived *T. cruzi* trypomastigotes at a 5:1 parasite/cell ratio in 0.5 mL of supplemented DMEM plus 2.5% FBS at 37°C in a 5% CO_2_ atmosphere, overnight. In the following day, monolayers were extensively washed to remove extracellular parasites and supplemented DMEM plus 2.5% FBS was added. In some experiments, the antioxidants NAC (20 mM; Sigma) or DFO (40 µM; Sigma) were diluted in sterile water and added or not to the cell cultures right after infection and washing step. After 72 h p.i., culture supernatants were collected for analysis and adherent NIH-3T3 cells were detached by treatment with phosphate buffer saline (PBS) containing 2 mM EDTA and 2% FBS, washed, and resuspended in supplemented DMEM plus 2.5% FBS. In some experiments, supernatants from either uninfected or infected NIH-3T3 were collected after 3 days of incubation, frozen and 50% of them were used with 50% of fresh medium to have a conditioned medium (CM) of *T. cruzi*-infected (Tc-CM) or non-infected (Ctrl-CM) conditions. The cellular numbers and viability were evaluated by the differential counting using Trypan blue solution (Sigma-Aldrich; 1:2). For assessment of parasite load, viable parasites, assessed as motile extracellular trypomastigotes released in culture supernatants, were counted in a Neubauer chamber.

### Proliferation Assay

To determine the proliferation of NIH-3T3 cells, carboxyfluorsecein diacetate succinimidyl ester (CFSE) staining was performed. NIH-3T3 cells (1 × 10^5^) were seeded in 75 cm^2^ culture flasks and after 5–7 days, cells were harvested by trypsinization and labeled with 5 µM CFSE (Molecular Probes, EUA) in PBS at 37°C for 20 min. Labeled cells were washed and seeded (5 × 10^5^ in six-well plates) with supplemented DMEM plus 2.5% FBS and left for adherence for 2 h. After this time, fresh culture medium containing tissue culture-derived *T. cruzi* trypomastigotes was added to the cells and cultured for 72 h at 37°C in a 5% CO_2_ atmosphere. After 72 h p.i., adherent NIH-3T3 cells were detached by treatment with PBS containing 2 mM EDTA and 2% FBS, washed and resuspended with FACS buffer (PBS containing 3% FBS and 0.02% sodium azide). Proliferation was evaluated using a FACSCalibur flow cytometer (BD). Fluorescence was compared with a T0 time point corresponding to cells incubated with CFSE and immediately analyzed. For analysis, Flow Jo software was used (TreeStar). To evaluate the relative frequency of proliferating (CFSE low) and non-proliferating (CFSE high) fibroblasts, we first gated the cells based on FSC-A versus SSC-A parameters.

### β-Galactosidase Assay

Senescence-associated β-galactosidase activity detectable at pH 6.0 was assayed according to Lee et al. (28). Briefly, equal numbers of cells were collected, washed, and resuspended in phosphate buffer (pH 6.0). Cells were lysed by freeze/thaw, and the lysates were centrifuged at 12,000 *g* for 7 min. The supernatants were mixed with 2-nitrophenyl-β-d-galactopyranoside (ONPG) (Sigma) (2.2 µg/µL) in 1 mM MgCl_2_ buffer. After incubation at 37°C for 12 h, 50 µL of 1 M sodium carbonate were added, and absorbance at 410 nm was measured in a plate spectrophotometer (SpectraMax M5 microplate reader).

### Cytokine Measurement

Supernatants from either uninfected or infected NIH-3T3, treated or not with NAC or DFO, were collected after 3 days of incubation and assayed for IL-6, IL-1β, TNF-α, MCP-1, and IL-10 cytokines by the sandwich immunoassay (ELISA), as recommended by the manufacturer (R&D Systems or eBioscience). The optical density was determined in a plate spectrophotometer (SpectraMax M5 microplate reader). The concentrations of cytokines were calculated from a standard curve of recombinant cytokines. Results are mean and SE of triplicate cultures.

### Nitric Oxide

Production of NO was assayed indirectly by quantification of nitrites accumulated in the supernatant of cultures by using the Griess colorimetric method described by Kwon et al. (44).

### Reactive Oxygen Species

Intracellular levels of ROS were measured by oxidation of nonfluorescent 2′,7′ dichlorofluorescin probe, delivered as diacetate form (DCFH-DA), to the fluorescent product 2′,7′ dichlorofluorescein (45). NIH-3T3 fibroblasts were plated in 96-well black plates and loaded for 20 min at 37°C with 10 µM DCFH-DA (Sigma-Aldrich). After the washing step with Hanks’ Balanced Salt Solution without phenol red (Gibco), fibroblasts were infected with tissue culture-derived *T. cruzi* trypomastigotes and treated or not with NAC or DFO, overnight. In the following day, monolayers were washed to remove extracellular parasites, fresh supplemented DMEM plus 2.5% FBS were added with or without the antioxidants NAC or DFO, for additional 3 days. Fluorescence was measured (529 nm excitation; 504 nm emission) from 2 to 72 h p.i. in a spectrophotometer (SpectraMax M5 microplate reader).

### Statistical Analyses

All statistical analyses were performed using Prism 6.0 software (GraphPad Software, La Jolla, CA, USA). All experiments shown are representative of at least two independent experiments with similar results. Normal distribution of data was determined using Kolmogorov–Smirnov test. Data with normal distributions were evaluated by unpaired Student’s two-tailed *t*-test. For the data that were not normally distributed, the non-parametric Mann–Whitney test was employed. For cells counting and parasite load analyses, data were log-transformed before statistical analyses. Data are expressed as the mean of technical replicates per treatment and SE. Differences with a *p* value <0.05 were considered significant.

## Results

### *T. cruzi* Infection Decreases the Number of NIH-3T3 Fibroblasts *In Vitro*

**Figure 1:**
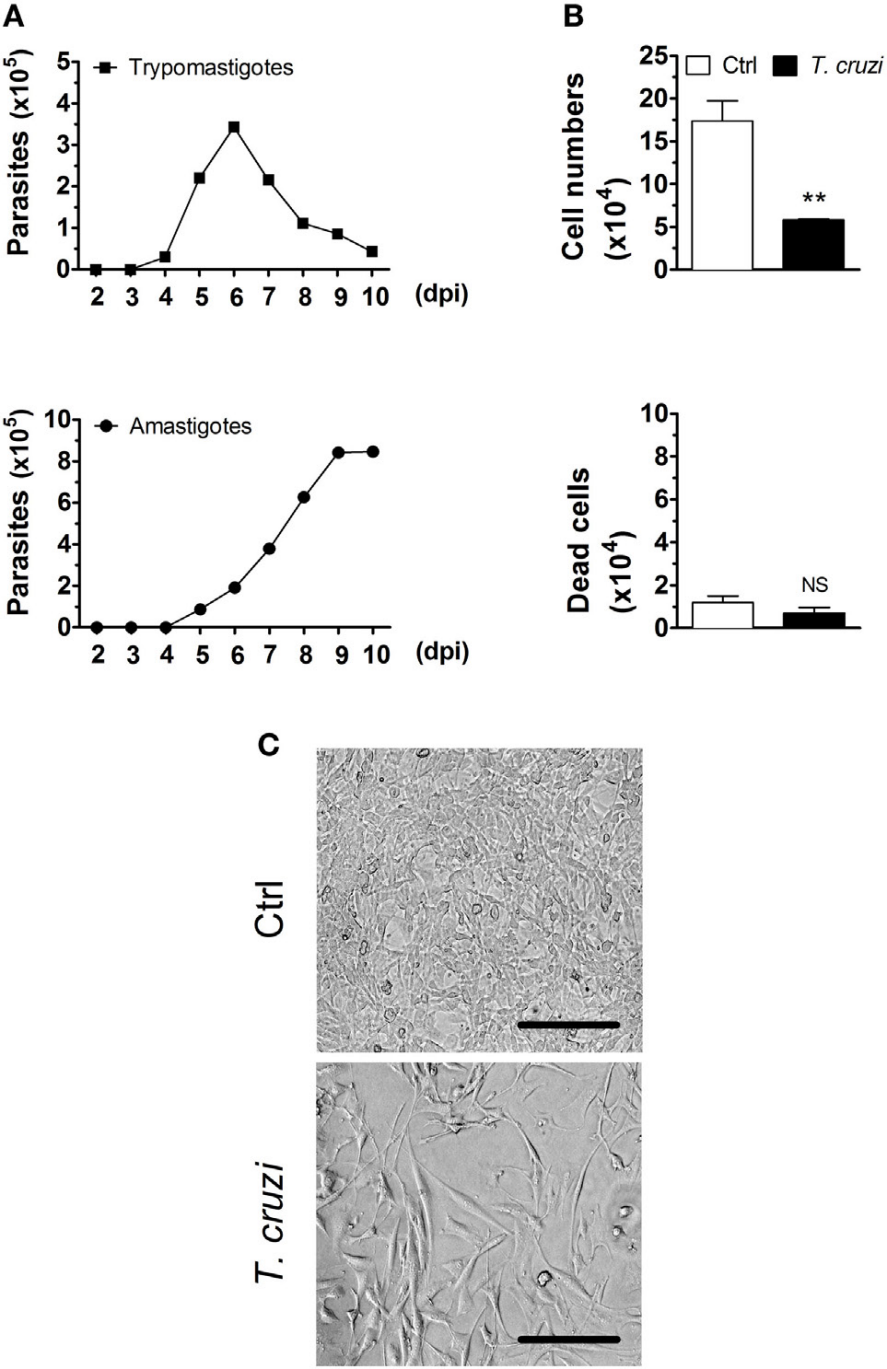
*Trypanosoma cruzi* infection decreases the number of fibroblasts in culture. NIH-3T3 fibroblasts were infected with culture-derived trypomastigotes of *T. cruzi* (MOI 5:1). **(A)** Assessment of extracellular parasitic load (trypomastigote and amastigote forms) in culture supernatants over 10 days of infection. **(B)** Quantification of the number of *T. cruzi*-infected or non-infected fibroblasts after 3 days post-infection in the optical microscope. Dead cells were evaluated by Trypan blue exclusion assay. **(C)** Photographs are representative of six randomly chosen fields and denote a confluent cell culture in the absence but not in the presence of *T. cruzi* infection (20× magnification). Scale bars are equal to 50 µm. Data are presented as the mean ± SE of three biological replicates. Data were log-transformed and analyzed by unpaired, two-tailed Student’s *t*-test. **p < 0.01; NS, not significant, *p* > 0.05. Data are representative of at least three independent experiments with similar results. Abbreviations: MOI, multiplicity of infection; dpi, days post-infection; Ctrl, control.

To investigate the initial events of *T. cruzi* infection, NIH-3T3 fibroblasts were infected and as expected, they released trypomastigotes into culture supernatants at day 4. At the day 6, we found the average of 10^5^ trypomastigotes released by each fibroblast (Figure 1A). We also observed an increasing of amastigote forms in the supernatants, which reflects a spontaneous differentiation of trypomastigotes to amastigotes, as previously described (46, 47). We next evaluated the effects of *T. cruzi* infection in NIH-3T3 fibroblasts by infecting these cells with culture-derived trypomastigotes overnight followed by a washing step to eliminate extracellular parasites. After 3 days p.i., cells were recovered from the plates and counted in the optical microscope. We found a remarkable reduction of the overall cell numbers in the infected compared to non-infected condition (Figure 1B, upper panel and Figure 1C). To investigate whether the reduced cell numbers in the infected condition were due to cell death, we further evaluated the numbers of dead cells by Trypan blue exclusion assay, and we observed equal numbers of dead cells in the infected and non-infected conditions (Figure 1B, lower panel and Figure 1C). By annexin V and 7AAD staining, we found that most of dead cells were apoptotic (data not shown). In addition, we observed equal numbers of detached cells in the supernatants in both conditions (data not shown). Infected fibroblasts displayed distinctive phenotypic alterations, including enlarged and flattened morphology (Figure 1C). Together, our data suggest that NIH-3T3 fibroblasts are not able to control the initial parasite replication, acting as a reservoir of *T. cruzi* at the early stages of infection; and that *T. cruzi* infection may interfere with the cellular proliferative capacity of fibroblasts.

### *T. cruzi* Infection Inhibits the Proliferation of NIH-3T3 Fibroblasts and Promotes Nuclei Accumulation of SAHF

**Figure 2:**
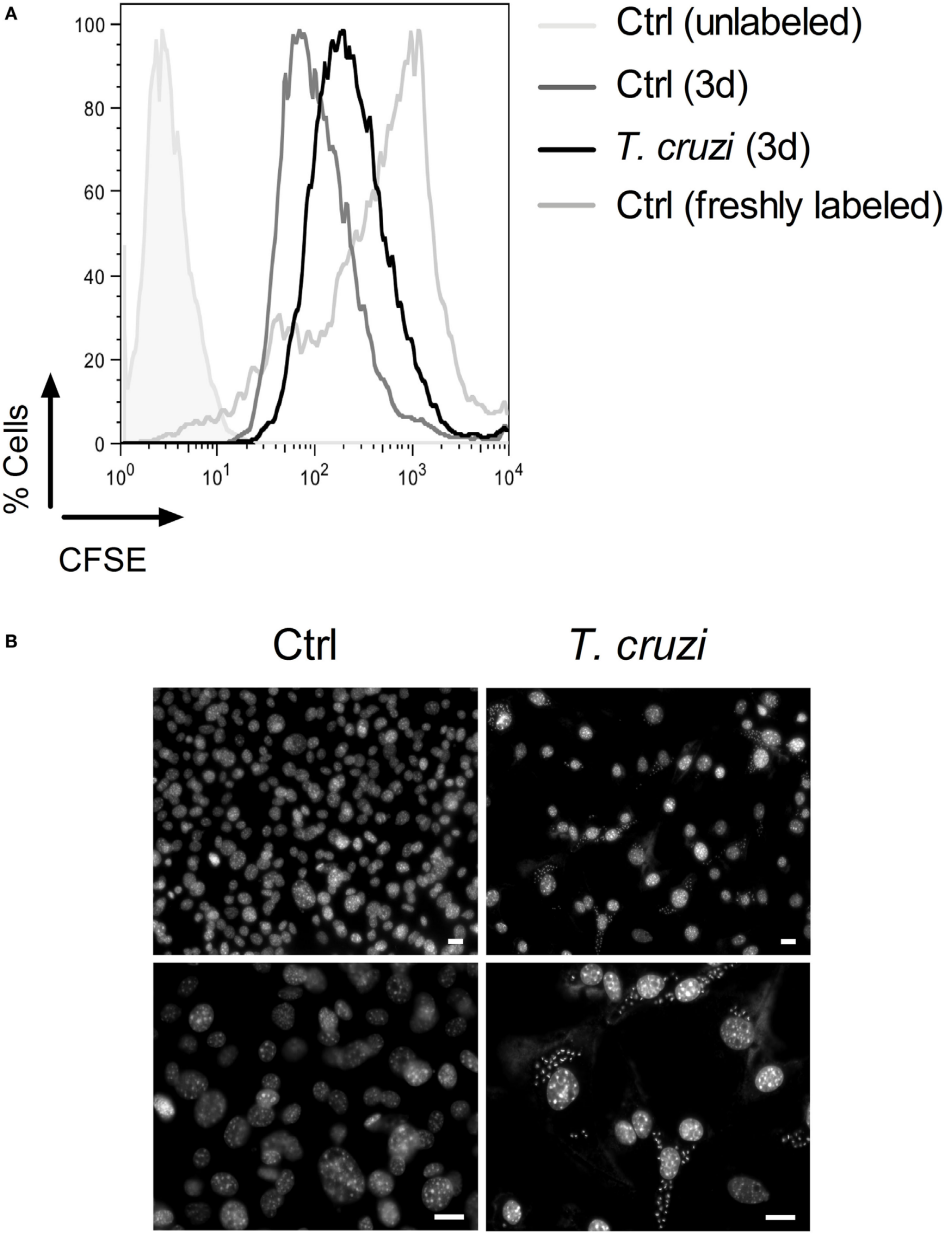
*Trypanosoma cruzi* infection inhibits fibroblasts proliferation and induces an accumulation of senescence-associated heterochromatin foci (SAHF). NIH-3T3 fibroblasts were infected with culture-derived *T. cruzi* trypomastigotes (MOI 5:1) overnight followed by a washing step to eliminate extracellular parasites. **(A)** Assessment of cellular proliferative capacity in CFSE-labeled fibroblasts 3 days (3d) post-infection and before infection (0d) by flow cytometry. Fibroblasts were first gated based on FSC-A versus SSC-A parameters, and then CFSE staining was analyzed. **(B)** *T. cruzi*-infected and non-infected NIH-3T3 fibroblasts stained with DAPI (upper panels, 20× magnification) 3 days post-infection. Enlarged images of DAPI staining are shown in the lower panels (40× magnification). The fluorescence images were obtained and photographed from multiple fields, by using an Axioplan II microscope (Zeiss) and image pro-plus software version 7.0.1. Scale bars are equal to 20 µm. Data are representative of at least three independent experiments with similar results. Abbreviations: MOI, multiplicity of infection; Ctrl, control; CFSE, carboxyfluorsecein diacetate succinimidyl ester.

To evaluate whether *T. cruzi* infection is able to interfere with the cell proliferation capacity, we infected CFSE-labeled fibroblasts with trypomastigotes and 3 days post-infection, we analyzed cell proliferation by flow cytometry (48). We observed that the fluorescence intensity of *T. cruzi*-infected NIH-3T3 fibroblasts stayed at high level while non-infected NIH-3T3 fibroblasts had a rapid decrease of fluorescence intensity, indicating that infection inhibited the proliferation of fibroblasts (Figure 2A). In addition, by DAPI staining, we found that the nuclei of *T. cruzi*-infected fibroblasts acquired the characteristic morphology of senescent cells, by showing an enlarged size and several bright, punctate SAHF (Figure 2B, upper and lower right panels) (26). By contrast, non-infected fibroblasts displayed a more uniform DAPI staining pattern (Figure 2B, upper and lower left panels). These data suggest that *T. cruzi* infection induces a senescent-like phenotype and alters NIH-3T3 fibroblasts proliferative ability.

### *T. cruzi* Infection Imprints a Senescent-Like Phenotype in NIH-3T3 Fibroblasts

**Figure 3:**
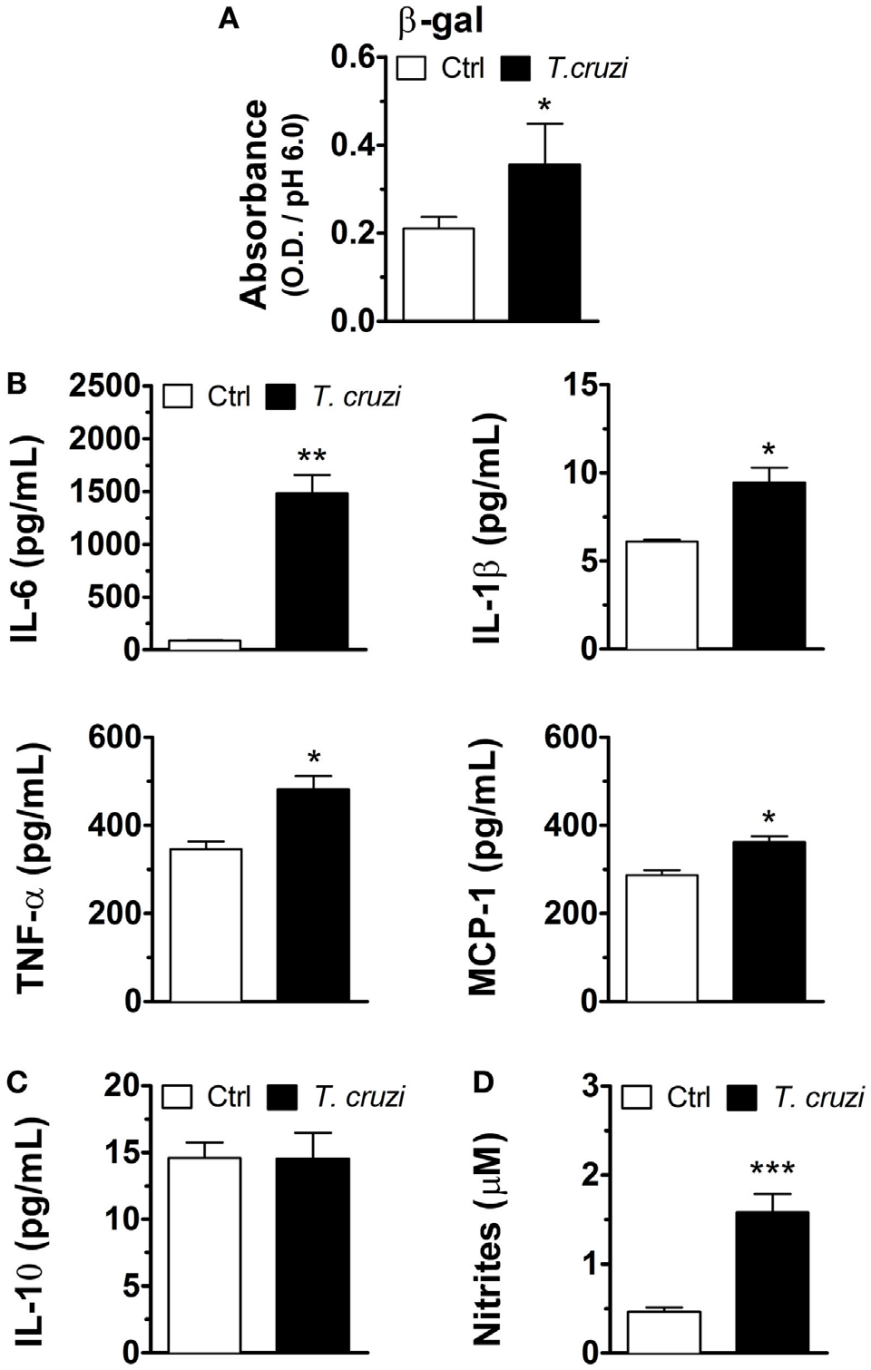
*Trypanosoma cruzi*-infected NIH-3T3 fibroblasts have characteristics of cellular senescence. NIH-3T3 fibroblasts were infected overnight with culture-derived *T. cruzi* trypomastigotes (MOI 5:1). **(A)** Evaluation of soluble SA-β-gal activity at pH 6.0 in cell lysates of *T. cruzi*-infected and non-infected fibroblasts 3 days post-infection. **(B,C)** Measurement of senescence-associated secretory phenotype (SASP) cytokines IL-6, TNF-α and IL-1β and chemokine MCP-1, as well as SASP non-related IL-10, in the 3 days post-infection culture supernatants by ELISA. **(D)** Evaluation of nitric oxide release by *T. cruzi*-infected and non-infected fibroblasts in culture supernatants 3 days post-infection by Griess reaction. Data are presented as the mean ± SE of at least six biological replicates and analyzed by **(A)** non-parametric Mann–Whitney test, and **(B–D)** unpaired, two-tailed Student’s *t*-test, *p < 0.05; **p < 0.01; ***p < 0.001; NS, statistically not significant, *p* > 0.05. Data are representative of at least three independent experiments with similar results. Abbreviations: MOI, multiplicity of infection; Ctrl, control; SA-β-gal, senescence-associated β-galactosidase.

Senescent cells are characterized by an increase in SA-β-gal activity (27, 28) and SASP (29, 30). To verify whether *T. cruzi*-infected NIH-3T3 fibroblasts have characteristics of cellular senescence, we first evaluated one of the hallmarks of cellular senescence, the soluble SA-β-gal activity, in cell lysates of *T. cruzi*-infected and non-infected fibroblasts. Our results showed that *T. cruzi* infection increased the activity of SA-β-gal measured at pH 6.0 in fibroblasts (Figure 3A). Furthermore, to verify whether *T. cruzi*-infected fibroblasts display SASP, we measured the levels of IL-6, IL-1β, TNF-α, and MCP-1 as well as non-SASP-related cytokine IL-10 in the culture supernatants 3 days p.i. We found that *T. cruzi*-infected fibroblasts produced markedly more IL-6, IL-1β, and TNF-α, as well as MCP-1 when compared to non-infected cells (Figure 3B). Moreover, we did not observe differences in the production of non-SASP cytokine IL-10 by infected and non-infected fibroblasts (Figure 3C). Furthermore, we found that *T. cruzi* infection induced the release of NO by NIH-3T3 fibroblasts in the culture supernatants (Figure 3D). Taken together these results indicate that *T. cruzi*-infected fibroblasts present typical senescent cells secretion profile.

### Antioxidants Inhibit the Induction of SASP and Control Parasite Growth in *T. cruzi*-Infected NIH-3T3 Fibroblasts

**Figure 4:**
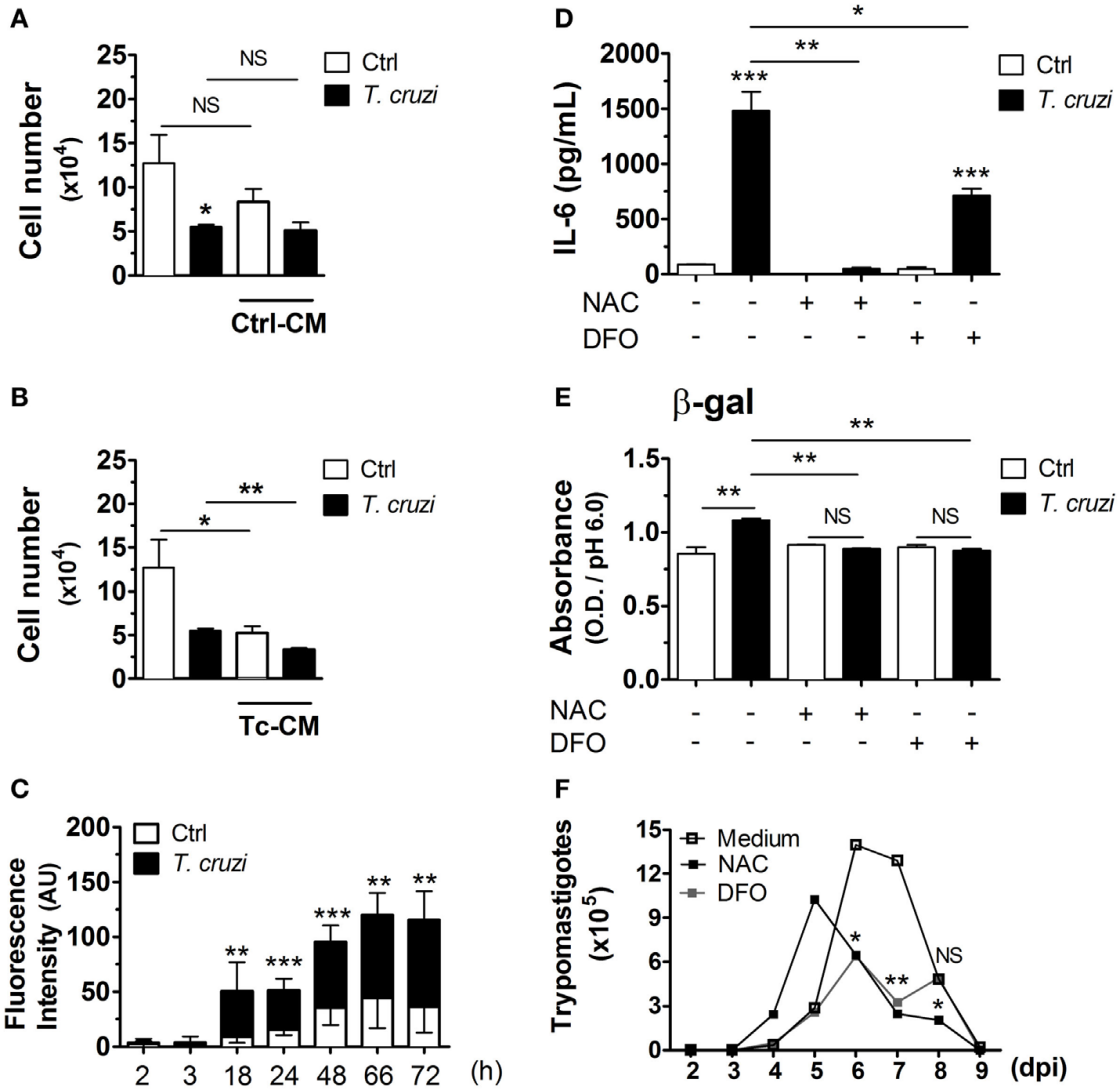
*Trypanosoma cruzi*-induced ROS is a potent inducer of SASP in NIH-3T3 fibroblasts. NIH-3T3 fibroblasts were infected overnight with culture-derived *T. cruzi* trypomastigotes (MOI 5:1). After washing step to eliminate extracellular parasites, cultures were treated with **(A)** 50% supernatants from non-infected or **(B)** *T. cruzi*-infected cultures (conditioned medium) plus 50% of fresh medium. After 3 days, the number of *T. cruzi*-infected or non-infected fibroblasts were quantified by optical microscopy. Dead cells were evaluated by Trypan blue exclusion assay. **(C–E)** NIH-3T3 fibroblasts were loaded with the probe DCFH-DA, washed and infected with *T. cruzi* concomitantly with addition of antioxidants NAC (20 mM) and DFO (40 µM) during infection period (MOI 5:1). After overnight infection, cultures were washed to eliminate extracellular parasites and cultured for additional 3 days. **(C)** Assessment of ROS accumulation after *T. cruzi* infection in NIH-3T3 fibroblasts by fluorescence. Results indicate arbitrary units of fluorescence. **(D)** Evaluation of IL-6 in culture supernatant by ELISA. **(E)** Evaluation of soluble SA-β-gal activity at pH 6.0 in cell lysates. **(F)** Assessment of extracellular parasitic load (trypomastigote forms) in culture supernatants over 10 days. Data are presented as the mean ± SE of at least six biological replicates and analyzed by unpaired, two-tailed Student’s *t*-test **(C,D)**, and non-parametric Mann–Whitney test **(E)**. For **(A,B,F)**, data were log-transformed and analyzed by Student’s *t*-test. *p < 0.05; **p < 0.01; ***p < 0.001; NS, statistically not significant, *p* > 0.05. Data are representative of at least three independent experiments with similar results. Abbreviations: MOI, multiplicity of infection; Ctrl, control; ROS, reactive oxygen species; NAC, *N*-acetylcysteine; DFO, deferoxamine; DCFH-DA, dichloro-dihydro-fluorescein diacetate; h, hours; ROS, reactive oxygen species; SASP, senescence-associated secretory phenotype; SA-β-gal, senescence-associated β-galactosidase.

To investigate whether the induction of senescent-like phenotype is due to autocrinally/paracrinally action of soluble factors released by *T. cruzi*-infected fibroblasts, NIH-3T3 cells were infected with culture-derived *T. cruzi* trypomastigotes (multiplicity of infection 5:1) overnight. After washing out extracellular parasites, cultures were treated with 50% supernatants from non-infected cultures (control) or *T. cruzi*-infected [control conditioned medium (Ctrl-CM) or *T. cruzi* CM (Tc-CM), respectively] plus 50% of fresh medium. After 3 days, the numbers of non-infected or *T. cruzi*-infected fibroblasts were quantified in the optical microscope. We found that the treatment with Ctrl-CM did not affect the numbers of fibroblasts in infected or non-infected cultures (Figure 4A). On the other hand, the treatment of non-infected cultures with Tc-CM for 3 days triggered a great reduction in the overall numbers of cells in a similar manner to the cultures infected with *T. cruzi* in the absence of Tc-CM treatment (Figure 4B). Interestingly, the treatment of *T. cruzi*-infected cultures with Tc-CM induced an even higher reduction of cell number compared to the Tc-CM non-treated cultures infected with *T. cruzi* (Figure 4B), suggesting that soluble factor(s) released by *T. cruzi*-infected fibroblasts control cellular proliferation and possible other senescence hallmarks in an autocrine/paracrine manner. One of the soluble factors potentially involved in the induction of senescence in infected fibroblasts are ROS, since their production has been implicated in DNA damage with a consequent control of cellular proliferation and senescence phenotype induction (49). We therefore assessed ROS accumulation after *T. cruzi* infection in NIH-3T3 fibroblasts using the fluorescent probe DCFH-DA. We observed an accumulation of ROS in infected fibroblasts starting 2 h p.i., and reaching a peak 72 h p.i. (Figure 4C). To determine whether the accumulation of ROS is critical for the induction of senescent-like phenotype by *T. cruzi* infection, we evaluated the soluble SA-β-gal activity and SASP cytokine IL-6 in cell lysates and supernatants, respectively, of *T. cruzi*-infected and non-infected fibroblasts, in the presence or absence of ROS scavengers NAC, a thiol compound that increases the levels of reduced glutathione, and DFO, an iron chelator that inhibits radical production. Briefly, cells were treated with the probe DCFH-DA 20 min followed by *T. cruzi* infection with addition of NAC or DFO during infection period. We confirmed that the addition of both antioxidants reduced ROS generation (Figure S1 in Supplementary Material). The treatment with antioxidants NAC or DFO during *T. cruzi* infection remarkably reduced SA-β-gal activity (Figure 4D) as well as the secretion of the SASP cytokines IL-6 (Figure 4E) by NIH-3T3 fibroblasts. On the other hand, the treatment with antioxidants did not restore the cellular proliferative capacity (data not shown), but reduced the number of released parasites by fibroblasts (Figure 4F). Together, our data suggest that *T. cruzi*-induced ROS are one of the main inducers of senescent-like phenotype in NIH-3T3 fibroblasts, which support the intracellular growth of *T. cruzi*.

## Discussion

The establishment of *T. cruzi* infection and therefore Chagas disease occurs when the parasites are internalized by phagocytic cells that are recruited to the vector’s bite sites. However, since these parasites have the ability to invade almost any cell type, even before reach immune cells, *T. cruzi* trypomastigotes may interact with non-immune cells, such as epithelial cells and fibroblasts in host skin and mucosal surfaces (11, 18). Thus, the early events of parasite–host interaction may play a fundamental role in development of immune responses and consequently in the outcome of chronic infection.

Although the interaction between *T. cruzi* and phagocytic cells such as macrophages is well established in the literature, little is known about *T. cruzi* infection in non-immune cells. To gain a clearer understanding of parasite–host interactions at early stages of Chagas disease, we examined cellular aspects of the *in vitro* infection of NIH-3T3 fibroblasts by *T. cruzi*. Our data reveal that NIH-3T3 fibroblasts are susceptible to *T. cruzi* infection with an earlier release of trypomastigotes to the culture medium (at the day 4) compared to macrophages (at the day 6) (50). In addition, we found a remarkable reduction in the overall numbers of cells in the infected compared to the non-infected fibroblasts, with equal numbers of dead (apoptotic) cells in both conditions, suggesting that *T. cruzi* infection may interfere with the cellular proliferative capacity of fibroblasts. In fact, regulating host cell cycle is one of the mechanisms used by many intracellular pathogens, including *T. cruzi*, to facilitate its replication and permanence within the organism (19–23). Furthermore, previous studies have suggested that *T. cruzi* infection impedes cell cycle progression in the host non-immune cells (22) and reprograms epithelial cells by regulating several genes involved in cellular defense, response to stress, and suppression of cell proliferation (11). In accordance, we found that *T. cruzi* infection alter NIH-3T3 fibroblasts phenotype and proliferative capacity, as verified by CFSE dilution assay.

Senescent cells do not proliferate, and are characterized by distinctive phenotypic alterations, including increased size and flattened morphology, enlarged and multinucleated nuclei (27, 51). Similarly, *T. cruzi*-infected NIH-3T3 fibroblasts acquired the characteristic senescent morphology showing enlarged and flattened cells with nuclei accumulation of SAHF, while non-infected fibroblasts displayed sharped morphology and a more uniform DAPI staining pattern. The induction of cellular senescence could provide an advantage or a disadvantage to the host. During homeostasis, the proliferative arrest avoids the development of cancer, since it prevents the propagation of damaged or stressed cells, which are in risk of neoplastic transformation (52, 53), for example. On the other hand, based on our *in vitro* studies, we believe that during Chagas disease, senescent fibroblasts, especially in the skin, become a long-term reservoir of parasites, which are released to infect resident and recruited cells. Besides the irreversible arrest of cell proliferation and the distinct heterochromatic structure (25, 26), senescent cells are characterized by an increased SA-β-gal activity due to the presence of more and bigger lysosomes (27, 51, 54). In accordance, NIH-3T3 fibroblasts infected with *T. cruzi* showed increased SA-β-gal activity, indicating that *T. cruzi* infection induces senescent-like phenotype in NIH-3T3 fibroblasts. Although increased SA-β-gal activity is a hallmark of senescent cells, its role in senescence induction and/or maintenance remains to be addressed.

All senescent cells are stably viable and continue to secrete large amounts of soluble factors, collectively named as SASP. SASP components are mainly growth factors, extracellular matrix remodeling enzymes, NO, chemokines, and proinflammatory cytokines, such as MCP-1, IL-6, TNF-α, and IL-1β (29–31). The role of SASP is to reinforce the senescence arrest in an autocrine and/or paracrine manner (55) able to alter the tissue microenvironment by recruiting and interacting with immune system cells, promoting the recognition and clearance of senescent cells (29, 30). We found that *T. cruzi*-infected fibroblasts display SASP, with markedly higher production of NO, MCP-1, IL-6, IL-1β, and TNF-α when compared to non-infected fibroblasts. Again, SASP can be beneficial or deleterious, depending on the biological context. In this sense, although this proinflammatory response could reflect defense mechanisms against the parasite, we cannot discard that SASP factors can be exploited by *T. cruzi* as a strategy for their survival and dissemination. Particularly, IL-1β, IL-6, TNF-α, and MCP-1 are efficient in triggering inflammation and recruitment of neutrophils, macrophages, and other immune cells (56–60), which can be infected by this parasite and spread the disease, since immune cells are mobile. In fact, the strategy for dissemination by neutrophil attraction and infection has been reported for *Leishmania* infection (61–63). Another SASP factor, NO, is an important cytotoxic and cytostatic factor in cell-mediated immune responses against many intracellular pathogens including *Leishmania* spp. and *Toxoplasma gondii* (64–67). However, its role in *T. cruzi* infection is controversial. Some studies support the role of NO in the control of *T. cruzi* infection (68, 69), while others demonstrate that NO is not required to eliminate these parasites (70, 71). In our model, although senescent infected fibroblasts produce NO, this factor seems not be important to eliminate intracellular *T. cruzi*. Moreover, the amount of NO released by *T. cruzi*-infected fibroblasts is very low when compared with macrophages (72, 73), and may not be sufficient to have a microbicidal effect on intracellular *T. cruzi*.

Cellular senescence occurs in culture and *in vivo* as a response to excessive extracellular or intracellular stress (74). A plethora of stresses can provoke cellular senescence, including dysfunction of mitochondria and oxidative stress (75). Notably, an important feature in the infection by *T. cruzi* is the oxidant stress response that is very relevant for the parasite’s pathogenesis. Although the oxidative stress generated after *T. cruzi* infection is a hallmark of professional phagocytic cells, it has been also demonstrated that this response is generated in non-phagocytic cells by many mechanisms, including an increased release of mitochondrial free radicals in cardiomyocytes (76), and in infected mice (77), as well as SASP cytokines signaling such as TNF (9). In agreement with that, we found an accumulation of ROS in infected fibroblasts. Many pathways could trigger cellular stress response characterized by increased ROS generation in *T. cruzi*-infected fibroblasts, including SAPK/JNK pathway, as previously shown by our group in macrophages infected by *Leishmania major* (78). In addition, the treatment with the antioxidants NAC and DFO during *T. cruzi* infection remarkably reduced the secretion of the most prominent SASP cytokine IL-6, and SA-β-gal activity by NIH-3T3 fibroblasts. On the other hand, the treatment with antioxidants did not restore the proliferative capacity of fibroblasts, suggesting that other factors than ROS control different aspects of cellular senescence induction in *T. cruzi*-infected fibroblasts. Importantly, we found a reduction in the number of trypomastigotes released by fibroblasts treated with DFO or NAC, which suggests that the presence of ROS during *T. cruzi* infection favor the intracellular growth of these parasites. Our data agree with the identified role of ROS in intracellular survival/growth of *Leishmania* and *T. cruzi* parasites (50, 78, 79). Based on that, we hypothesize that *in vivo* treatment with antioxidants could control the parasitemia and perhaps, the spread of Chagas disease. Furthermore, we found that soluble factors released by *T. cruzi*-infected fibroblasts can control cellular proliferation and possible other senescence hallmarks in a paracrine manner in non-infected fibroblasts through CM experiments. However, we neither identify the exact factor(s) responsible for the cell arrest in infected fibroblasts nor the role of these soluble factors modulating the function of other cells such as epithelial cells, and resident and recruited immune cells. Besides their effects in fibroblasts, we hypothesize that SASP factors such as IL-6 and NO released by senescent cells can modulate the functions of neighborhood macrophages, dendritic cells (DCs), T cells as well as epithelial cells, and keratinocytes. In fact, it has been described that IL-6 induces epidermal cell proliferation and thickening of *stratum corneum*, increasing skin protection from infection (80), which is probably occurs during skin manifestations of Chagas disease.

In macrophages, IL-6 promotes M2 polarization (81, 82) and blocks DCs maturation (83, 84). Similarly, NO suppresses DCs maturation as well as their capacity to activate naïve T cells (85–87), inhibits proliferation and production of IFN-γ by T cells (88), and promotes the differentiation of Treg cells (89). All these factors may favor *T. cruzi* survival and permanence in the host during acute phase of Chagas disease. Future studies using Tc-CM in macrophages and/or DCs cultures need to be performed to evaluate whether factors released by *T. cruzi*-induced senescent fibroblasts could modulate microbicidal function, antigen presentation, migration properties, and induction of senescence in these phagocytic immune cells.

In summary, our results provide new information regarding the early cellular responses to *T. cruzi* infection that may be relevant to the establishment of Chagas disease. We showed that fibroblasts are susceptible to *T. cruzi* infection and they are not able to control the initial parasite replication. Moreover, *T. cruzi* infection inhibits cellular proliferation and induces a senescent-like phenotype in fibroblasts, which may allow more time for *T. cruzi* to replicate and produce its intracellular nests. Thus, senescent-like fibroblasts act as a reservoir of *T. cruzi* at the initial stages of infection. The exact role of the senescence in the control or progression of Chagas disease will be further investigated, thus contributing to our better understanding of this complex chronic disease. Our findings provide new insight into the mechanisms by which intracellular *T. cruzi* infection influences the host cells, leading to pathogenicity; and could be important to define new therapeutic interventions to treat Chagas disease.

## Author Contributions

KG-P, DN, GD, and AF designed experiments and analyzed data. KG-P, DN, and AC-F performed experiments. GD and AF supervised the experiments. CF-d-L and AM contributed reagents/analysis tools. ML contributed reagents/analysis tools and to the critical revision of the manuscript. GD conceived the research. AF wrote the manuscript. All authors read and approved the submitted version of the manuscript.

## Conflict of Interest Statement

The authors declare that the research was conducted in the absence of any commercial or financial relationships that could be construed as a potential conflict of interest.
