# Supplementary material for: Trypanosoma cruzi Infection Induces Cellular Stress Response and Senescence-Like Phenotype in Murine Fibroblasts
Source: Front Immunol. 2018 Jul 9;9:1569. doi: 10.3389/fimmu.2018.01569 (PMC6047053; doi:10.3389/fimmu.2018.01569)
Supplement: Supplementary file 1 [file image_1.PDF]

Supplementary Material

Trypanosoma cruzi infection induces cellular stress response and senescence in murine fibroblasts

Kamila Guimarães-Pinto, Danielle Nascimento, Antonia Corrêa-Ferreira, Celio G. Freire-de-Lima, Alexandre Morrot, Marcela F. Lopes, George A. DosReis, Alessandra A. Filardy*

*** Correspondence:** Alessandra A. Filardy: filardy@micro.ufrj.br

1 Supplementary Figures

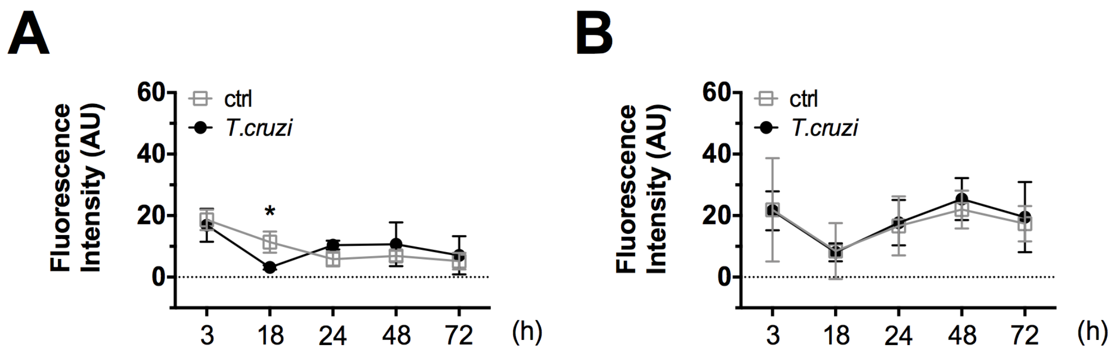

Supplementary Figure 1. Antioxidants inhibit ROS production in *T. cruzi*-infected NIH-3T3 fibroblasts. Fibroblasts were pretreated with the antioxidants (**A**) NAC (20 mM) and (**B**) DFO (40 mM) for 1 hour and then loaded with the probe DCFH-DA, washed and infected with *T. cruzi* concomitantly with another addition of antioxidants during infection period (MOI 1:5). After overnight infection, cultures were washed to eliminate extracellular parasites and cultured for additional 3 days. Graphs represent the assessment of ROS accumulation by fluorescence and the results indicate arbitrary units of fluorescence. Data are representative of at least three independent experiments with similar results, and are presented as the mean ± SE of three biological replicates and analyzed by unpaired, two-tailed Student's *t* test, *, $p < 0.05$; NS, statistically not significant, $p > 0.05$. MOI, multiplicity of infection; Ctrl, control; ROS, reactive oxygen species; NAC, N-acetyl cysteine; DFO, deferoxamine; DCFH-DA, dichloro-dihydro-fluorescein diacetate; h, hours.
